# Pleurodesis Induction in Rats by Copaiba (*Copaifera multijuga* Hayne) Oil

**DOI:** 10.1155/2014/939738

**Published:** 2014-06-09

**Authors:** Fernando Luiz Westphal, Mauro Canzian, Fabio Alessandro Pieri, Alfredo Coimbra Reichl, Paulo Manuel Pêgo-Fernandes, Luis Carlos Lima, Valdir F. Veiga-Junior

**Affiliations:** ^1^Hospital Universitário Getúlio Vargas, Universidade Federal do Amazonas, Avenida Apurinã 4, Praça 14 de Janeiro, 69020-170 Manaus, AM, Brazil; ^2^Hospital das Clínicas da Faculdade de Medicina, Instituto do Coração (InCor), Universidade de São Paulo, Avenida Dr. Enéas de Carvalho Aguiar, 44, Jardim Nossa Senhora Aparecida, 05403-900 São Paulo, SP, Brazil; ^3^Departamento de Ciências Básicas da Saúde, Universidade Federal de Juiz de Fora, Câmpus Governador Valadares, Rua Israel Pinheiro, 2000, Bairro Universitário, 35010-177 Governador Valadares, MG, Brazil; ^4^Instituto de Ciências Exatas, Universidade Federal do Amazonas, Avenida Rodrigo Otávio 6200, Coroado, 69.080-005 Manaus, AM, Brazil

## Abstract

This study aims to assess and compare copaiba oleoresin of *Copaifera multijuga* and 0.5% silver nitrate for the induction of pleurodesis in an experimental model. Ninety-six male Wistar rats were divided into three groups: control (0.9% saline solution), copaiba (copaiba oil), and silver nitrate (0.5% silver nitrate). The substances were injected into the right pleural cavity and the alterations were observed macroscopically and microscopically at 24, 48, 72, and 504 h. The value of macroscopic alterations grade and acute inflammatory reaction grade means was higher in the 24 h copaiba group in relation to silver nitrate. Fibrosis and neovascularization means in the visceral pleura were higher in 504 h copaiba group in relation to the silver nitrate group. The grade of the alveolar edema mean was higher in the silver nitrate group in relation to the copaiba group, in which this alteration was not observed. The presence of bronchopneumonia was higher in the 24 h silver nitrate group (*n* = 4) in relation to the copaiba group (*n* = 0). In conclusion, both groups promoted pleurodesis, with better results in copaiba group and the silver nitrate group presented greater aggression to the pulmonary parenchyma.

## 1. Introduction

Pleurodesis is a well-established treatment for patients with spontaneous pneumothorax or malignant pleural effusion of multiple etiologies. The objective of pleurodesis is to promote the symphysis of the parietal and visceral pleura by means of induction of fibrosis with the consequent obliteration of the pleural space to prevent the accumulation of either air or fluid in the pleural cavity [1–3].

The ideal agent for pleurodesis has not yet been found. Several substances have been studied both clinically and experimentally, such as ethanolamine oleate, diazepam, erythromycin, macrolides, quinolones, povidone-iodine, and quinacrine, among others [4–7]. All of these studies intend to find an agent with the following characteristics: (1) efficient, (2) widely available, (3) of low cost, and (4) of few side effects. Today, the most commonly used agents are talc, instilled in the pleural cavity by either aspersion or dilution in saline solution, tetracycline derivatives (minocycline and doxycycline), and bleomycin [8]. In the 1980s, tetracycline was the preferred agent for chemical pleurodesis. However, due to cessation of manufacturing, it is no longer available for such an application. Bleomycin has a very high cost and is less efficient in pleurodesis when compared to other medications [9].

Silver nitrate is a substance with irritating, caustic, astringent, and disinfectant properties; it is of low cost and is easy to handle and sterilize and instill in the pleural cavity. Its use for pleurodesis has been increasing, especially after its utilization at smaller concentrations, which has decreased the painful side effects [10]. However, these side effects, in cases that required larger amounts, boost new research in the search for new therapeutic alternatives for inducing pleurodesis.

The Amazon is a main reserve of natural products on the planet, and its population makes wide empirical use of the medicinal properties of these substances due to distrust and the high cost of industrialized medications. Copaiba oil, one of the most popularly used natural products, is known for its antimicrobial, anticancer, antiseptic, and healing effects, among others [11–23]. It can be taken orally or used topically to reestablish the normal conditions of the mucosa affected by diseases, especially the urethral, vesical, bronchial, and tonsillar mucosa [24]. This oil is composed exclusively of diterpenes and sesquiterpenes, which are naturally produced substances with several biological activities already established in the literature [24, 25].

The effect of copaiba oil injected into the peritoneal and pleural cavities of rats, contrary to what was expected, produced an intense, universal inflammatory reaction with the formation of firm adherences associated with the thickening of the serosas [26, 27]. Based on these findings, we intend to use copaiba oil as an irritating substance, with a possible effect for the induction of pleurodesis.

This study aims to assess and compare copaiba oleoresin of* Copaifera multijuga* and 0.5% silver nitrate for the induction of pleurodesis in an experimental model.

## 2. Material and Methods

In an experimental, randomized study, 96 adult, male Wistar rats (*Rattus norvegicus)* weighing on average 200 g, of the same lineage, were used, from the Instituto Nacional de Pesquisas da Amazônia (INPA) Bioterium. The study was approved by the Ethics Committee for Animal Experimentation of Federal University of Amazonas in September 16, 2008.

The rats were divided into three groups: control (0.9% saline solution), copaiba (pure copaiba oil), and 0.5% silver nitrate, in which each group was composed of 32 animals. After the instillation of the substances in the pleural cavity, each group was further divided into four subgroups of eight rats according to the time of sacrifice, at 24, 48, 72, and 504 h (21 days).

**Figure 1:**
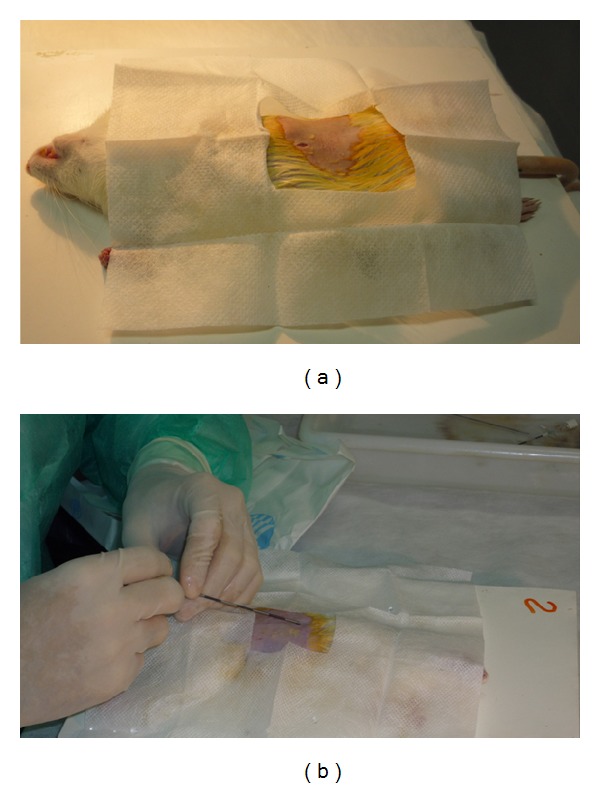
Procedures applied in rats to evaluate the induction of pleurodesis. (a) Rat under subcutaneous anesthesia and local asepsis with topical povidone-iodine, in the subxiphoid region; a small incision can be observed. (b) The injection in the right pleural cavity with epidural anesthesia needle using the transdiaphragmatic approach.

The copaiba oil was extracted from the trunk of a tree from the* Copaifera multijuga* species located at Forest Reserve Ducke, Manaus, AM, Brazil. The main constituents of the oil were as previously reported in oil C21-1 in Barbosa, Medeiros [28]. Sesquiterpenes were identified by relative retention index, mass spectrum, and comparison with standards. Diterpenes were isolated and NMR data was compared with the literature [28]. The 0.5% silver nitrate and 0.9% sterile saline solution with an electrolyte content of sodium and chloride of 154 mEq/l were obtained commercially. A volume of 0.4 mL of 0.5% silver nitrate or 0.2 mL of copaiba oil or 1 mL of saline solution was injected into the pleural cavity of the animals. After the rats were weighed, trichotomy was performed at the incision site and, under subcutaneous anesthesia with chloral hydrate (0.2 to 0.3 mL), a 5 mm incision was made, preceded by local asepsis with topical povidone-iodine, in the subxiphoid region (Figure 1(a)). The substances were injected with an epidural anesthesia needle using the transdiaphragmatic approach, in the right pleural cavity (Figure 1(b)). It was determined that rats, with weight less than 200 g, would receive injection of 0.35 mL of substances and over 200 g rats would receive injection of 0.4 mL. This dosage was based on the weight proportion of an average male adult human of 70 kg and an average male of 198.9 g, with this result multiplied by two, due to double pleural cavity of this animal.

At the preestablished time periods (24, 48, 72, and 504 h), the animals were euthanized through the administration of a toxic dose of chloral hydrate (0.8 mL/100 g) in order to induce cardiorespiratory arrest.

Immediately after the sacrifice, a median incision of the thorax and abdomen was performed to observe the macroscopic alterations that were classified as described by Tonietto et al. [29], according to the modification of the pleural cavity and pulmonary parenchyma, in the following grades:

 grade 0: no macroscopic alteration;
 grade 1: presence of exudate, no fibrin reaction or adherences;
 grade 2: presence of exudate, with the presence of mild adherences;
 grade 3: presence of exudate, with the presence of firm adherences;
 grade 4: absence of exudate, incarcerated lung.

After macroscopic analysis, the right lung and the parietal pleura of the right anterolateral chest wall were removed and placed in recipients containing an adequate volume of 10% buffered formalin and randomly numbered from “1” to “96.” After fixation, a longitudinal cut of the right lung was performed with the removal of a longitudinal segment of the anterior chest wall, approximately 1.5 cm long and 0.4 cm wide, containing soft parts, cartilaginous costal segments, and parietal pleura. These samples were submitted to automated histological processing and put in paraffin blocks, at the Pathology laboratory at Incor. In the histological analysis, 4 *μ*m thick sections were stained using the hematoxylin-eosin method and analyzed by a pathologist who was blinded to the medication previously injected. The histological alterations observed were considered separately in relation to the visceral pleura, parietal pleura, and pulmonary parenchyma and according to the components of the inflammatory process they were classified as acute and chronic. Thus, acute inflammation was described by predominance of edema, fibrin deposition, associated or not with inflammatory infiltrate, composed predominantly of neutrophils. In chronic inflammation, there was a predominance of fibroblastic proliferation, vascular neoformation, and presence of infiltrate composed predominantly of mononuclear cells and fibrosis, indicated by collagen matrix deposition. These factors were semiquantified both together and separately, and scores were then attributed to them as follows: “0” for the absence of the referred alteration, “1” for alterations of low intensity, “2” for alterations of moderate intensity, and “3” for intense alterations, according to the methodology established by Hurewitz et al. [30].

Qualitative data was obtained by calculating the simple and relative absolute frequencies. Regarding the quantitative data, the mean and the standard deviation (SD) were calculated when there was normal distribution of data, and median, first (*Q*_1_) and third quartile (*Q*_3_) when the distribution of data was not normal. Fisher's exact test was used in the association analysis, whereas Student's* t*-test was used to compare the means of the grades of the macroscopic and microscopic alterations, since there was a normal distribution of data. Kruskal-Wallis' nonparametric test was used to compare the weight difference. The software used in the analysis was Epi-Info 3.4.3 for Windows developed and distributed by CDC (http://wwwn.cdc.gov/epiinfo/), and the level of significance used in the tests was 5%.

## 3. Results

The injection of substances in the animals did not produce symptoms and did not require analgesia, and there were no deaths during the experiment. The variation in weight measured before and after the experiment showed no relation to the substance injected. The mean of weight variations in the animals after intrapleural instillation was −11.48 ± 15.00 mg, −18.05 ± 17.85 mg, −31.38 ± 25.79 mg, and 61.82 ± 56.15 mg after 24, 48, 72, and 504 h, respectively.

**Figure 2:**
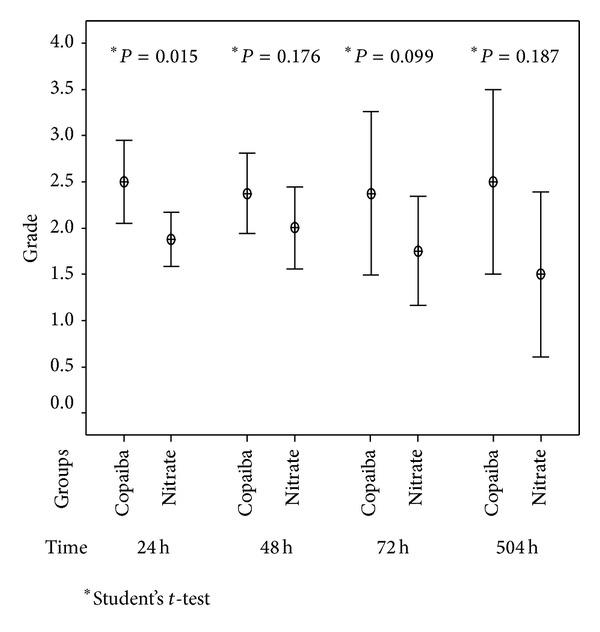
Pleurodesis induction by copaiba oil and silver nitrate in rats. Distribution according to mean of grade of macroscopic inflammatory reaction according to the time of experiment in relation to the groups after.

Under macroscopic and microscopic evaluations, all animals of the control group were of grade 0. No significant changes in macroscopic or microscopic evaluations were found in the control group. Greater macroscopic inflammatory reactions were observed in the rats that received copaiba oil, in all the times of sacrifice, when compared to the silver nitrate group, with statistical significance in the 24-hour group (*P* < 0.05) (Figure 2).

**Figure 3:**
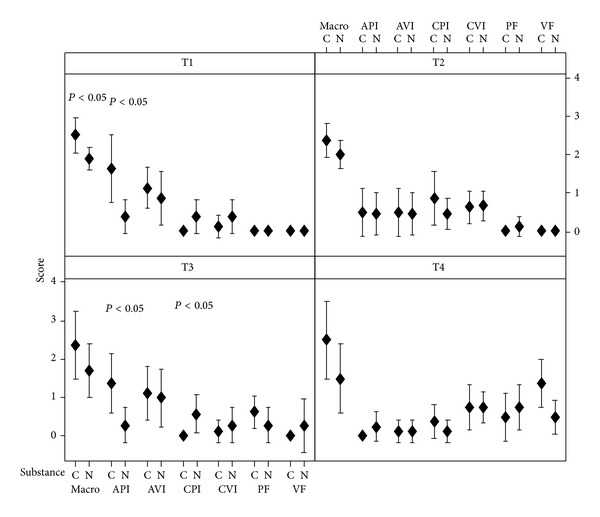
Distribution of means of scores presented in macroscopic and microscopic analysis after treatment with the substances, in different times. C: copaiba oil; N: silver nitrate; macro: macroscopic; API: acute parietal inflammation; AVI: acute visceral inflammation; CPI: chronic parietal inflammation; CVI: chronic visceral inflammation; PF: parietal fibrosis; VF: visceral fibrosis; T1: 24 h; T2: 48 h; T3: 72 h; T4: 504 h; *P* < 0.05 was considered as significant, presenting difference between the treatments.

Macroscopic and microscopic changes related to the process of inflammation and acute and chronic fibrosis, both in the parietal and visceral pleura, in different times, are detailed in Figure 3. It demonstrates that copaiba oil presents more obvious macroscopic changes every time, with microscopic correspondence at 72 h and 504 h. It is observed that at times 24 h and 48 h there was no development of fibrosis in both groups, which was more evident at times 72 h and 504 h (Figure 3).

Regarding the analysis of the acute inflammatory reaction of the parietal pleura, there was a more intense reaction in the copaiba group at 24 and 72 h (*P* = 0.01 and 0.008, resp.), when compared to the silver nitrate group, as presented in Table 1. In visceral pleura, regarding the same reaction, we observed a greater inflammation effect in the copaiba group at 24 and 72 h, however, without statistical significance (Table 1).

Regarding chronic parietal inflammation, a greater grade of fibrosis was observed in the 72 h silver nitrate group with statistical significance (*P* = 0.019) when compared to copaiba. There was no difference between the two substances in relation to the visceral pleura (Table 1).

Fibrosis of the visceral and parietal pleura was only observed at 72 and 504 h with increased reaction in the copaiba group to visceral fibrosis at 504 h (*P* = 0.017), as presented in Table 2.

Neovascularization, one of the main indicators of inflammation, was found at a greater grade in the 504 h copaiba group (1.50 ± 1.07), when compared to the silver nitrate group (0.34 ± 0.52; *P* = 0.018) (Table 2).

Edema of the parietal pleura was observed in all cases with the two substances, without statistical significance. In the visceral pleura, edema was more intensely present in the 24 h silver nitrate group when compared to the copaiba group (*P* = 0.041). The alveolar edema difference was more evident in the 24 h 0.5% silver nitrate group, with statistical significance (*P* = 0.003), when compared to the copaiba oil group. In the 48 and 72 h groups, the presence of edema was more frequent in the silver nitrate group (Table 2).

Bronchopneumonia was observed in four rats of the 24 h silver nitrate group, considered statistically significant (*P* = 0.038) when compared to copaiba oil. In the copaiba oil group, only two rats presented this characteristic, in the 48 and 504 h groups, respectively.

## 4. Discussion

Despite the indication of copaiba oil as anti-inflammatory by the literature [24], a previous work identified an intense inflammatory reaction in the pleural surface when the copaiba oil was applied topically for the treatment of empyema (data not published). Thus, based on this finding, the possibility of testing this phytotherapic as a sclerosing agent was identified. Copaiba oil was used previously, experimentally, in the pleural cavity of rats with the development of great pleural reactions and multiple adherences [26]. The authors described adherences and early lung incarceration at 48 and 72 hours, however, with a higher dose of copaiba oil, which probably caused higher mortality when compared to the current study [26]. In another experimental study, in which copaiba oil was instilled in the peritoneal cavity, the macroscopic alterations observed were similar to those found in the pleura, thus showing that copaiba oleoresin triggers aggression in the serosa with a corresponding formation of adherences in the process to repair the injury [27].

In this study, the macroscopic alterations were observed in both groups; however, they were more evident in the copaiba group for all of the times analyzed, showing that oleoresin when in contact with the pleural surface causes a greater pleural reaction than that induced by silver nitrate. The most frequent findings were adherences and pleural thickenings. The ventral region contained more alterations, and it is the area of greater contact with the substance in the pleura of rats. Similarly, Kennedy et al. [31], who used talc to induce pleurodesis in rabbits, also observed a greater number of alterations in the same region. Despite talc being the most used substance to pleurodesis induction, in this study, we opted to use silver nitrate because it is easier in the experimental procedure and is equally effective.

The efficacy of the 0.5% silver nitrate solution was tested in other experiments using rabbit models, with a similar effect of pleurodesis when compared to 35 mg/kg tetracycline [32] and superior effect when compared to talc diluted in saline solution at 400 mg/kg [10]. In addition to these studies, silver nitrate already has a well-defined clinical application, as described by Marcheix et al. [33], who showed a 98.9% success rate in pleurodesis when instilled by video-assisted thoracoscopy, however, at a 1% concentration.

The mechanism of the development of pleurodesis after the injection of the substance on the pleural surface has not been fully understood. The first alterations are the descaling of mesothelial cells and the development of pleural effusion with characteristics of exudate. After this, the effusion evolved to pleural adhesions and, in the reparative phase, we observed inflammatory reaction to injury and the regeneration of damaged cells, the migration of conjunctive tissue, the synthesis of extracellular protein matrix, and collagenization, determining a greater consistency of the pleural symphysis. Tissue healing evolution is complex and involves several interrelated processes, including acute inflammatory reaction to the injury on the first day and regeneration of cellular damage with cell migration of the connective tissue to the damaged area between the third and fifth days. Afterwards, protein synthesis of the extracellular matrix begins, a process that can last days or weeks making the healing tissue firmer [10, 31].

The action of copaiba oleoresin in the pleural space can be similar to that observed in most of the substances used for pleurodesis, but the mechanism that leads to the induction of pleurodesis has not been defined. According to Westphal et al. [26], the microscopic analysis of the pleura showed four times greater pleural thickening in the copaiba oil group with statistical significance than the substances compared in that study, crajiru and povidone-iodine, which were mild irritants. In this study, there was pleural thickening, however, at lower intensity, probably due to the reduced dose of copaiba oil injected in the pleural cavity.

The alterations in pulmonary parenchyma due to the use of silver nitrate were described by Vargas et al. [34], who witnessed alveolar collapse and signs of hemorrhage and discrete edema in the first month of follow-up. After the second month, minimal alterations similar to those in the talc group were observed. In this study, we observed a greater reaction in the animals of the silver nitrate group, evidenced by alveolar and visceral pleura edema, in addition to the development of bronchopneumonia in this group when compared to the copaiba group.

Neovascularization associated with the formation of pleural adherences does not differ from its diverse origins since they are similar in the cases of inflammatory and neoplastic processes. The formation of this new tissue is conditioned to the presence of new vascularization as observed in rabbits, in which the presence of neovascularization was observed both in the parietal and the visceral pleura [30]. The importance of neovascularization in the formation of pleurodesis was demonstrated by Guo et al. [35], who reduced it significantly when using the endothelial growth inhibition antibody in rabbits, a substance that inhibits the action of the transforming growth factor (TGF) and is known as a sclerosing agent. In this study, more evident neovascularization was observed in the 504 h time period in the copaiba oil group, when compared to the silver nitrate group, which shows greater effect of copaiba oil in the production of pleurodesis.

The use of rats in experiments to check the effects of pleurodesis can be achieved successfully as observed by Marchi et al. [36]. Several advantages were found in comparison to other animals, among them the easy handling and low cost of maintenance of these animals. In our experiments, we observed that pleurodesis occurred in both groups, thus showing that the use of rats as an animal model in the induction of pleurodesis can be carried out experimentally, allowing the study of pleuropulmonary alterations of pleurodesis.

With respect to chemical constitution, the major constituents in the oil of* C. multijuga* were copalic acid, caryophyllene oxide, and caryophyllene [28], which are common in oils of various species of copaiba trees [25]. Based on this, it is suggested that the action presented in this study may be common in many oils that have the same chemical profile, taken from many other species of* Copaifera*. However, the pharmacological effect of the oleoresin cannot be attributed to just one constituent, because the constituents present in oleoresin may interact synergistically in the promotion of the activity observed [13, 25].

## 5. Conclusions

In conclusion, this study has shown that intrapleural injection of copaiba oil and 0.5% silver nitrate produced pleurodesis; however, the macroscopic alterations and acute inflammation were more evident in the copaiba oil group. During the work period, greater pulmonary alterations occurred in the 0.5% silver nitrate group when compared to copaiba oil. Thus, both groups promote pleurodesis; however, the silver nitrate group presented greater aggression to the pulmonary parenchyma, introducing copaiba oil as a potential treatment for the induction of pleurodesis with milder side effects. However, additional studies are needed to confirm the activity of copaiba oil, identify its potential active compounds in inducing pleurodesis, and evaluate the possible side effects of its use to thereby define its usefulness in patients who have malignant diseases.

## Figures and Tables

**Table 1:** Distribution according to the mean of the grade of microscopic alterations according to parietal and visceral inflammation in relation to the groups.

*T*	Acute inflammation		P*	Chronic inflammation		P*
	Copaiba	Silver nitrate		Copaiba	Silver nitrate	
	Mean ± SD	Mean ± SD		Mean ± SD	Mean ± SD	
Parietal pleura						
24 h	1.63 ± 1.06	0.38 ± 0.52	***0.010***	0.00 ± 0.00	0.38 ± 0.52	0.060
48 h	0.50 ± 0.78	0.50 ± 0.78	0.999	0.88 ± 0.83	0.50 ± 0.53	0.303
72 h	1.38 ± 0.92	0.25 ± 0.46	***0.008***	0.00 ± 0.00	0.50 ± 0.53	***0.019***
504 h	0.00 ± 0.00	0.25 ± 0.46	0.149	0.38 ± 0.52	0.13 ± 0.35	0.278

Visceral pleura						
24 h	1.12 ± 0.64	0.88 ± 0.83	*0.513 *	0.13 ± 0.35	0.38 ± 0.52	0.278
48 h	0.50 ± 0.78	0.50 ± 0.78	0.999	0.63 ± 0.52	0.63 ± 0.52	0.999
72 h	1.13 ± 0.83	0.88 ± 0.83	*0.559 *	0.13 ± 0.35	0.38 ± 0.52	*0.278 *
504 h	0.13 ± 0.35	0.13 ± 0.35	0.999	0.75 ± 0.71	0.75 ± 0.46	0.902

*Student's *t*-test. Value of *P* in bold italic indicates statistical difference at 5%. *T*: time of euthanasia.

**Table 2:** Distribution according to the mean of the scores of the variables analyzed.

Variable	24 h	48 h	72 h	504 h
	Mean ± SD	Mean ± SD	Mean ± SD	Mean ± SD
Parietal fibrosis				
Copaiba oil	—	—	0.63 ± 0.52	0.50 ± 0.78
Silver nitrate	—	—	0.38 ± 0.52	0.75 ± 0.71
P*	—	—	0.350	0.506
Visceral fibrosis				
Copaiba oil	—	—	0.00 ± 0.00	1.38 ± 0.74
Silver nitrate	—	—	0.25 ± 0.71	0.50 ± 0.54
P*	—	—	—	***0.017***
Parietal neovascularization				
Copaiba oil	—	0.25 ± 0.46	0.63 ± 0.52	0.50 ± 0.53
Silver nitrate	—	0.50 ± 0.53	0.63 ± 0.52	0.63 ± 0.74
P*	—	0.334	0.999	0.705
Visceral neovascularization				
Copaiba oil	0.00 ± 0.00	—	0.25 ± 0.46	1.50 ± 1.07
Silver nitrate	0.13 ± 0.35	—	0.62 ± 0.92	0.34 ± 0.52
P*	0.334	—	0.319	***0.018***
Parietal edema				
Copaiba oil	0.50 ± 0.53	0.75 ± 0.46	1.25 ± 0.71	0.63 ± 0.74
Silver nitrate	0.38 ± 0.52	0.88 ± 0.35	1.00 ± 0.53	0.63 ± 0.74
P*	0.642	0.554	0.438	0.999
Visceral edema				
Copaiba oil	0.38 ± 0.52	0.87 ± 0.64	1.62 ± 0.92	1.50 ± 0.76
Silver nitrate	0.88 ± 0.35	1.25 ± 0.46	1.38 ± 0.52	1.38 ± 0.52
P*	***0.041***	0.201	0.513	0.705
Fibroblastic proliferation				
Parietal pleura				
Copaiba oil	—	0.50 ± 0.76	0.62 ± 0.52	0.75 ± 1.03
Silver nitrate	—	0.63 ± 0.52	1.25 ± 0.46	0.63 ± 0.52
P*	—	0.705	***0.023***	0.764
Fibroblastic proliferation				
Visceral pleura				
Copaiba oil	—	0.13 ± 0.35	1.12 ± 0.64	1.12 ± 0.83
Silver nitrate	—	0.25 ± 0.46	0.75 ± 0.89	0.75 ± 0.46
P*	—	0.554	0.349	0.285
Alveolar edema				
Copaiba oil	0.00 ± 0.00	0.25 ± 0.71	0.00 ± 0.00	—
Silver nitrate	1.50 ± 1.20	0.50 ± 0.53	0.50 ± 0.76	—
P*	***0.003***	0.438	0.082	—

*Student's *t*-test. Value of *P* in bold italic indicates statistical difference at 5%.

## References

[1] Light RW, Vargas FS (1997). Pleural sclerosis for the treatment of pneumothorax and pleural effusion. *Lung*.

[2] Dikensoy O, Light RW (2005). Alternative widely available, inexpensive agents for pleurodesis. *Current Opinion in Pulmonary Medicine*.

[3] Rodriguez-Panadero F, Janssen JP, Astoul P (2006). Thoracoscopy: general overview and place in the diagnosis and management of pleural effusion. *European Respiratory Journal*.

[4] Teixeira LR, Vargas FS, Carmo AO, Silva LMMF, Marchi E, Light RW (1998). Effectiveness of ethanolamine oleate as a pleural sclerosing agent in rabbits. *Respiration*.

[5] Miller Q, Meschter C, Neumaster T (2007). Comparison of pleurodesis by erythromycin, talc, doxycycline, and diazepam in a rabbit model. *Journal of Surgical Education*.

[6] Olivares-Torres CA, Laniado-Laborín R, Chávez-García C, León-Gastelum C, Reyes-Escamilla A, Light RW (2002). Iodopovidone pleurodesis for recurrent pleural effusions. *Chest*.

[7] Stiksa G, Korsgaard R, Simonsson BG (1979). Treatment of recurrent pleural effusion by pleurodesis with quinacrine: comparison between instillation by repeated thoracenteses and by tube drainage. *Scandinavian Journal of Respiratory Diseases*.

[8] Lee P, Sun L, Lim CK, Aw SE, Colt HG (2010). Selective apoptosis of lung cancer cells with talc. *European Respiratory Journal*.

[9] Muduly DK, Deo S, Subi TS, Kallianpur AA, Shukla NK (2011). An update in the management of malignant pleural effusion. *Indian Journal of Palliative Care*.

[10] Vargas FS, Teixeira LR, Antonangelo L (2001). Experimental pleurodesis in rabbits induced by silver nitrate or talc: 1-year follow-up. *Chest*.

[11] Drumond MR, Castro RD, Almeida RV, Pereira MS, Padilha WW (2004). Comparative study *in vitro* of the antibacterial activity from phytotherapeutic products against cariogenical bacteria. *Pesquisa Brasileira em Odontopediatria e Clínica Integrada*.

[12] Gonçalves AL, Alves-Filho A, Menezes H (2005). Comparative study on antimicrobial activity of some native tree extracts. *Arquivos do Instituto Biológico*.

[13] Izumi E, Ueda-Nakamura T, Veiga VF, Pinto AC, Nakamura CV (2012). Terpenes from *Copaifera* demonstrated *in vitro* antiparasitic and synergic activity. *Journal of Medicinal Chemistry*.

[14] Lima SRM, Veiga VF, Christo HB, Pinto AC, Fernandes PD (2003). *In vivo* and *in vitro* studies on the anticancer activity of *Copaifera multijuga* hayne and its fractions. *Phytotherapy Research*.

[15] Maciel MAM, Pinto AC, Veiga VF, Grynberg NF, Echevarria A (2002). Medicinal plants: the need for multidisciplinary scientific studies. *Quimica Nova*.

[16] Pacheco TA, Barata LE, Duarte MC (2006). Antimicrobial activity of copaiba (*Copaifera spp*) balsams. *Revista Brasileira de Plantas Medicinais*.

[17] Pieri FA, José RM, Galvãeo NN, Nero LA, Moreira MAS (2010). Antimicrobial activity of autoclaved and non autoclaved copaiba oil on *Listeria monocytogenes*. *Ciencia Rural*.

[18] Pieri FA, Martins Mussi MC, Fiorini JE, Scatamburlo Moreira MA, Schneedorf JM (2012). Bacteriostatic effect of copaiba oil (*Copaifera officinalis*) against *Streptococcus mutans*. *Brazilian Dental Journal*.

[19] Pieri FA, Mussi MC, Fiorini JE, Schneedorf JM (2010). Clinical and microbiological effects of copaiba oil (*Copaifera officinalis*) on dental plaque forming bacteria in dogs. *Arquivo Brasileiro de Medicina Veterinaria e Zootecnia*.

[20] Pieri FA, Silva VO, Souza CF, Costa JCM, Santos LF, Moreira MAS (2012). Antimicrobial profile screening of two oils of copaifera genus. *Arquivo Brasileiro de Medicina Veterinaria e Zootecnia*.

[21] Pieri FA, Souza CF, Costa JCM (2011). Inhibition of *Escherichia coli* from mastitic milk by copaiba oil. *Semina: Ciencias Agrarias*.

[22] Dos Santos AO, Ueda-Nakamura T, Dias Filho BP, Veiga VF, Pinto AC, Nakamura CV (2008). Antimicrobial activity of Brazilian copaiba oils obtained from different species of the *Copaifera* genus. *Memorias do Instituto Oswaldo Cruz*.

[23] Nakamura CV, Dos Santos AO, Ueda-Nakamura T, Dias Filho BP, Da Veiga Junior VF (2012). Copaiba oil: an alternative to development of new drugs against leishmaniasis. *Evidence-Based Complementary and Alternative Medicine*.

[24] Veiga VF, Pinto AC (2002). The *Copaifera* L. genus. *Quimica Nova*.

[25] Leandro LM, de Sousa Vargas F, Barbosa PCS, Neves JKO, da Silva JA, da Veiga-Junior VF (2012). Chemistry and biological activities of terpenoids from copaiba (*Copaifera spp*.) oleoresins. *Molecules*.

[26] Westphal FL, Lima LC, Guimarães RAG, Sousa RFS, Couto SB, Nakajima SR (2007). Evaluation of the pleuropulmonary alterations after injection of copaiba oil, aqueous extract of crajiru and iodine PVP in the pleural space of mice. *Revista do Colégio Brasileiro de Cirurgiões*.

[27] Souza Júnior OG, Guimarães Neto HP, Pinto NT, Santos MT, Carvalho RA (2002). Macroscopic findings in peritoneal cavity of rats after injection of copaiba oil. *Revista Paraense de Medicina*.

[28] Barbosa PCS, Medeiros RS, Sampaio PTB, Vieira BG, Wiedemann LSM, Veiga- VF (2012). Influence of abiotic factors on the chemical composition of copaiba oil (*Copaifera multijuga* Hayne): soil composition, seasonality and diameter at breast height. *Journal of the Brazilian Chemical Society*.

[29] Tonietto T, Pilla ES, Madke GR (1999). Experimental pleural empyema in rats: effect of the intrapleural administration of dextran 40 during the fibrinopurulent stage. *Jornal de Pneumologia*.

[30] Hurewitz AN, Lidonicci K, Wu CL, Reim D, Zucker S (1994). Histologic changes of doxycycline pleurodesis in rabbits. Effect of concentration and pH. *Chest*.

[31] Kennedy L, Harley RA, Sahn SA, Strange C (1995). Talc slurry pleurodesis: pleural fluid and histologic analysis. *Chest*.

[32] Vargas FS, Teixeira LR, Silva LMMF, Carmo AO, Light RW (1995). Comparison of silver nitrate and tetracycline as pleural sclerosing agents in rabbits. *Chest*.

[33] Marcheix B, Brouchet L, Renaud C (2007). Videothoracoscopic silver nitrate pleurodesis for primary spontaneous pneumothorax: an alternative to pleurectomy and pleural abrasion?. *European Journal of Cardio-Thoracic Surgery*.

[34] Vargas FS, Antonangelo L, Capelozzi V (2002). Lung damage in experimental pleurodesis induced by silver nitrate or talc: 1-year follow-up. *Chest*.

[35] Guo YB, Kalomenidis I, Hawthorne M, Parman KS, Lane KB, Light RW (2005). Pleurodesis is inhibited by anti-vascular endothelial growth factor antibody. *Chest*.

[36] Marchi E, Vargas FS, Acencio MMP (2007). Pleurodesis: a novel experimental model. *Respirology*.

